# Implementation of a Composite Reference Standard Methodology in the Greek National Malaria Reference Center, Insights from a Seven-Year Experience

**DOI:** 10.3390/pathogens15080782

**Published:** 2026-07-23

**Authors:** Nikolaos Tegos, Leonidas Georgalis, Anastasia Bimpa, Anastasia Panagopoulou, Stavroula Beleri, Vasilios Papavasilopoulos, Annita Vakali, Chrisovaladou Kefaloudi, Danai Pervanidou, Christos Hatzichristodoulou, Eleni Patsoula

**Affiliations:** 1 Laboratory for the Surveillance of Infectious Diseases, Department of Public Health Policy, School of Public Health, University of West Attica, 115 21 Athens, Greece; nmpimpa@uniwa.gr (A.B.); apanagopoulou@uniwa.gr (A.P.); smpeleri@uniwa.gr (S.B.); vpapavasilopoulos@uniwa.gr (V.P.); epatsoula@uniwa.gr (E.P.); 2 Laboratory of Hygiene and Epidemiology, Department of Medicine, University of Thessaly, Papakyriazi 22, 412 22 Larisa, Greece; leonidasgeorgalis@gmail.com (L.G.); xhatzi@uth.gr (C.H.); 3 National Public Health Organization (NPHO), 151 23 Athens, Greece; a.vakali@eody.gov.gr (A.V.); c.kefaloudi@eody.gov.gr (C.K.); d.pervanidou@eody.gov.gr (D.P.)

**Keywords:** malaria, *Plasmodium* species, identification, imported, indigenous, microscopy, rapid diagnostic test, polymerase chain reaction

## Abstract

Background: Malaria is currently ranked as an important public health issue in Greece despite its initial eradication back in 1974. Given that the clinical picture is often nonspecific, the need for the implementation of targeted laboratory methodologies is imperative to ensure prompt *Plasmodium* identification and diagnosis. Methods: A total of 780 clinical samples from 763 suspected malaria cases from the National Malaria Reference Center were analyzed within a seven-year period (2017–2023). A composite laboratory reference standard methodology, including rapid diagnostic testing, microscopy, and polymerase chain reactions, was implemented for the purposes set by the current study in a group of 532 samples. A multivariable statistical analysis was implemented for the interpretation of the extracted data. Results: Microscopy and PCR successfully identified *P. falciparum* (111 vs. 131) and *P. vivax* (95 vs. 95) clinical samples. In addition, microscopy successfully identified selected cases of *P. ovale* (16) and *P. malariae* (nine), with current cases being confirmed by targeted, species-level real-time PCR protocols. Conclusions: The current study highlighted the importance of microscopic examination in malaria diagnostic procedures for *Plasmodium* species identification and typing. The combination of microscopy and PCR ensures a credible laboratory approach for submicroscopic clinical cases and patients with prior antimalarial treatment. This composite laboratory reference standard methodology is recommended by the Greek Malaria Reference Center for prompt identification of *Plasmodium* species in suspected malaria clinical cases.

## 1. Introduction

Malaria, a parasitic disease transmitted through the bite of an infected female Anopheles mosquito, constitutes one of the most important public health issues. There are five known *Plasmodium* species causing malaria in humans, namely *Plasmodium falciparum*, *Plasmodium vivax*, *Plasmodium malariae*, *Plasmodium ovalecurtisi*, *Plasmodium ovalewallikeri*, and *Plasmodium knowlesi* [1]. According to the World Health Organization (WHO), for the year 2024 alone, an estimated 282 million cases and 610,000 deaths were recorded. The most vulnerable target group continues to be children under five years of age, accounting for over 75% of the total number of malaria deaths in the WHO African Region for the same time period [2].

The clinical picture of malaria is characterized by a vast range of nonspecific symptoms, such as fever, fatigue, abdominal pain, myalgia, nausea, vomiting, diarrhea, chills, and headache, all of which can lead to misdiagnosis [3]. Febrile travelers returning from malaria-endemic countries must be promptly checked for malaria, since a delay in *P. falciparum* diagnosis can lead to complications [4]. The fact that—apart from cases from malaria-endemic countries—a progressively increasing number of imported and locally acquired cases has been recorded in non-endemic countries during the last few decades further necessitates the need for the implementation of targeted and reliable diagnostic approaches.

Greece has been malaria-free since 1974; since then, 20–110 imported cases have been recorded annually, mainly involving travelers or immigrants from malaria-endemic countries. In 2025, 47 laboratory-confirmed malaria cases were recorded, of which 44 were classified as imported and two as locally acquired, while one case had an undetermined importation status. Fifty-five percent (55%) of the imported cases concerned immigrants. The majority (93%) of imported cases were infected in Sub-Saharan countries (mainly Nigeria, Sudan, Cameroon, Sierra Leone, Ethiopia and Tanzania). A total of 10 patients exhibited complicated malaria (nine from *P. falciparum* and one from *P. ovale* and *P. malariae* co-infection) [5].

A malaria diagnosis relies on rapid and accurate identification of the causative *Plasmodium* species in order to administer the proper therapeutic regimen. Microscopic examination, performed using Giemsa-stained thin and thick blood smears for *Plasmodium* species, is implemented for species identification and parasitemia count and is considered the gold standard for a malaria diagnosis [6]. Results can be obtained within a few hours of receiving the clinical sample, and blood smears need to be repeated while the patient is febrile in case of an initial negative result [7,8]. Rapid diagnostic tests (RDTs) have emerged as a supplement to microscopy, especially in settings in which microscopy is not available [9]. Results can be obtained within 15 min, without the need for an experienced user. These lateral-flow immunochromatographic tests on nitrocellulose strips can detect antigens for *P. falciparum* or non-*P. falciparum* infections in blood samples. Molecular methods have emerged in an attempt to overcome the limitations posed by microscopy in malaria diagnosis. Compared with microscopy, molecular methods are 10-fold more sensitive, having a detection limit of about 0.2–6 parasites/μL (depending upon the assay used and the *Plasmodium* species under detection) [10]. They comprise a vast range of different methodological approaches, the most common of which is polymerase chain reaction (PCR) and its variations (i.e., multiplex PCR, nested PCR, real-time PCR). In cases when microscopy cannot successfully diagnose malaria at the *Plasmodium* species level, nested and multiplex PCR protocols can provide an accurate identification [11]. The Malaria Reference Center (MRC) in Greece has accredited three different methods, namely microscopy, PCR and RDTs, for laboratory investigations of suspected malaria cases. Furthermore, the suggested workflow is strengthened by the use of real-time PCR protocols targeted to the species level for the verification of suspected malaria cases caused by *P. ovale*, *P. malariae*, and *P. knowlesi*.

The aim of this seven-year retrospective study is to provide insights regarding the principles followed by the MRC to ensure credible identification to the species level of suspected malaria cases, regardless of the incriminated *Plasmodium* species.

## 2. Materials and Methods

### 2.1. Samples

Whole blood samples from febrile patients constitute the optimum type of clinical sample for malaria diagnosis. A stained slide is either provided by the referral hospital or prepared by the skilled microscopists at MRC in order to be examined for *Plasmodium* sp. presence and for subsequent verification at the species level. Due to the life-threatening risk of malaria infections and the public heath importance of malaria transmission risk, all blood samples received are accepted for laboratory testing, and all diagnosis methods are reimbursed from the National Public Health Organization (NPHO) (providing free-of-charge diagnosis for the patient). Upon arrival at the MRC, the temperature of the sample is checked and noted along with the day of blood collection (if provided), since preservation conditions can affect the results of the implemented testing protocols. Following the standard operating procedures (SOPs) of the MRC, the appropriate documents are filled out, and the laboratory personnel initiates the malaria testing protocols described thereafter.

For the purposes of the current study, a total number of 780 samples from 763 unique patients were collected within a seven-year period (2017–2023).

### 2.2. Microscopy

Blood samples are processed immediately upon reception in the laboratory. Two (2) thin films for each sample are made on appropriately marked, clean, grease-free microscopy slides and left to air dry. The dried slides are fixed with Methanol (PanReac Applichem, Darmstadt) and stained with a 10% Giemsa solution for 15 min, rinsed with tap water, and left to air dry completely. Each slide is examined with light microscopy using an oil immersion objective lens for the presence of *Plasmodium* species. Two (2) microscopists examine the slides independently. To declare a slide as negative, at least 200 objective fields are examined with no *Plasmodium* sp. stage observed. If during examination, even one distinct form (i.e., gametocyte or trophozoite) of *Plasmodium* species is identified, the sample is considered positive. Identification at the species level is accomplished by using the morphological criteria described in the WHO malaria guidelines [12]. When *P. falciparum* is identified, the microscopists also calculate the relative parasitemia.

### 2.3. Rapid Diagnostic Tests

The MRC uses the Malaria P.f/Pan Ag Rapid test (Abbott Bioline, Abbott Park, IL, USA), which is the same one used by referring hospitals and is supplied by NPHO. This test offers differential diagnosis between *P. falciparum* and the species *P. vivax*, *P. ovale*, and *P. malariae* (indication Pan) in human whole blood samples. The test is based on the detection of HRP2 (Histidine-rich protein 2), specific to *P. falciparum*, and pLDH (*Plasmodium* lactate dehydrogenase), specific to *Plasmodium* species. This form of cassette testing, stable at temperatures 1–40 °C, displays 99.7% Relative Sensitivity and 99.5% Relative Specificity for P.f (HRP2) and a Relative Sensitivity of 95.5% and Relative Specificity of 99.5% for Pan (pLDH), respectively. A sample of 5 μL of whole blood is placed in the appropriate well, and three drops of lysis buffer are added to the buffer well according to manufacturer’s instructions. The test is read after 15–30 min. This rapid test includes an internal control band; the test is invalid and needs to be repeated if the control fails to show a clear band. No such incident was observed during the testing procedures. If only the control band appears, the test is negative. If the band for the control and the P.f. protein appear, the test is positive for *P. falciparum*. If the control and the Pan bands are observed, the test is positive for non-*P. falciparum* infection, without specifying which *Plasmodium* species is implicated. Finally, if all three bands (control, P.f. and Pan) appear, we may have a *P. falciparum*-positive finding or a case of mixed infection. To ascertain the validity of the results, the MRC tests each new kit before using it with known positive samples as an internal quality control.

The MRC has also ordered a second RDT from Healgen Scientific (Houston, TX, USA), as certain cases of clinical samples found to be positive for *P. ovale* during microscopy are declared to be negative by the respective RDT from Abbott. For the purposes of the current study, both the referring hospitals and the MRC have used the RDT from Abbott.

### 2.4. Molecular Methods

An in-house multiplex PCR protocol was implemented, amplifying simultaneously and in a single reaction *P. falciparum*- and *P. vivax*-positive samples as previously described [13]. This protocol was slightly modified; namely, a quantity of 3 μL of genomic DNA corresponded to a total reaction volume of 30 μL. The amplified PCR products displayed product sizes of 346 bp and 266 bp for *P. falciparum* and *P. vivax*, respectively. This particular molecular approach has been selected and accredited by MRC since 2015.

### 2.5. Statistical Analysis and Agreement Metrics

The total number of clinical samples of the study was collected within a seven-year period (2017–2023), and a subset was investigated using three different methodologies: light microscopy, the rapid diagnostic test (RDT), and the polymerase chain reaction (PCR). The polymerase chain reaction (PCR) served as the reference standard throughout the analysis, reflecting its superior analytical sensitivity and ability to detect submicroscopic parasitemia. To ensure that every performance metric was computed on an identical denominator and that comparisons between methods were directly interpretable, the analysis was restricted to samples with a valid result from all three methods. Entries recorded as “not done”, “unsuitable”, or “doubtful” were therefore excluded, yielding 551 eligible samples.

The analysis was further restricted to the diagnostic categories of primary interest: negative, *P. falciparum*, *P. vivax*, and the RDT pan-antigen result (“Pan”). Mixed infections (*P. falciparum*/*P. vivax* co-infections) and the less frequent species *P. ovale* and *P. malariae* were excluded, as these were represented by too few cases to support meaningful comparison and fell outside the detection range of the RDT and the PCR approaches. This restriction removed a further 19 samples, resulting in a final analytic sample number of 532 samples.

The RDT results are classified as “Pf” (*P. falciparum*-specific HRP2 antigen), “Pan” (pan-malarial pLDH antigen, indicative of a non-falciparum infection), or “negative”. Because *P. ovale* and *P. malariae* were excluded from the analytic statistical analysis, every remaining “Pan” result corresponded to a *P. vivax* infection. The “Pan” result was therefore treated as *P. vivax* in all calculations, while retaining its original “Pan” label in tables and figures, so that the RDT readout remains transparent to the reader.

Descriptive statistics summarized the distribution of diagnostic outcomes and of the key categorical variables. Associations between PCR-based positivity and demographic or sampling characteristics were assessed using the chi-square test of independence, with statistical significance defined as *p* < 0.05.

Binary diagnostic performance (positive versus negative) was evaluated for microscopy and RDT against PCR. Sensitivity, specificity, positive predictive value (PPV), and negative predictive value (NPV) were calculated for each method, with 95% confidence intervals (CIs) derived using the Wilson score method, which maintains reliable coverage for proportions close to 0 or 1. Cohen’s unweighted kappa (κ) quantified chance-corrected agreement, with 95% CIs obtained by percentile bootstrap resampling (2000 iterations; fixed random seed for reproducibility). McNemar’s test with continuity correction assessed whether discordant result pairs were distributed symmetrically, thereby identifying any systematic directional bias in misclassification. Kappa values were interpreted according to the framework of McHugh (2012) [14], in which a κ of 0.00–0.20 denotes no agreement, 0.21–0.39 denotes minimal agreement, 0.40–0.59 denotes weak agreement, 0.60–0.79 denotes moderate agreement, 0.80–0.90 denotes strong agreement, and above 0.90 denotes an almost perfect agreement; this framework was preferred over that of Landis and Koch (1977) [15], as it applies stricter thresholds that were considered more appropriate for health-related diagnostic studies.

Species-level agreement between each method and PCR was quantified using unweighted Cohen’s kappa, appropriate for nominal categories given that *Plasmodium* species carry no inherent ordinal ranking. With the “Pan” result mapped to *P. vivax*, all three methods shared a common three-category classification (negative, *P. falciparum*, *P. vivax*), and confusion matrices were constructed accordingly. Confidence intervals for species-level κ were again obtained by percentile bootstrap resampling (2000 iterations).

Species-specific diagnostic performance was further characterized using a one-versus-rest approach, in which each target species formed the positive class and all remaining results formed the negative class. This was applied to *P. falciparum* and *P. vivax* for microscopy, and to *P. falciparum* (HRP2 line) and *P. vivax* (pan-pLDH “Pan” line) for the RDT. Sensitivity, specificity, PPV, and NPV with 95% Wilson CIs were computed for each comparison, and differences in sensitivity between species were tested using the chi-square test or Fisher’s exact test, the latter applied when any expected cell count fell below five.

All analyses were performed in R (version 4.5.1; R Core Team). Data management and tabulation used the dplyr, tidyr, janitor, and purrr packages. Agreement statistics were computed with irr. Figures were produced with ggplot2 and assembled using patchwork.

### 2.6. Structured Diagnostic Workflow

**Figure 1:**
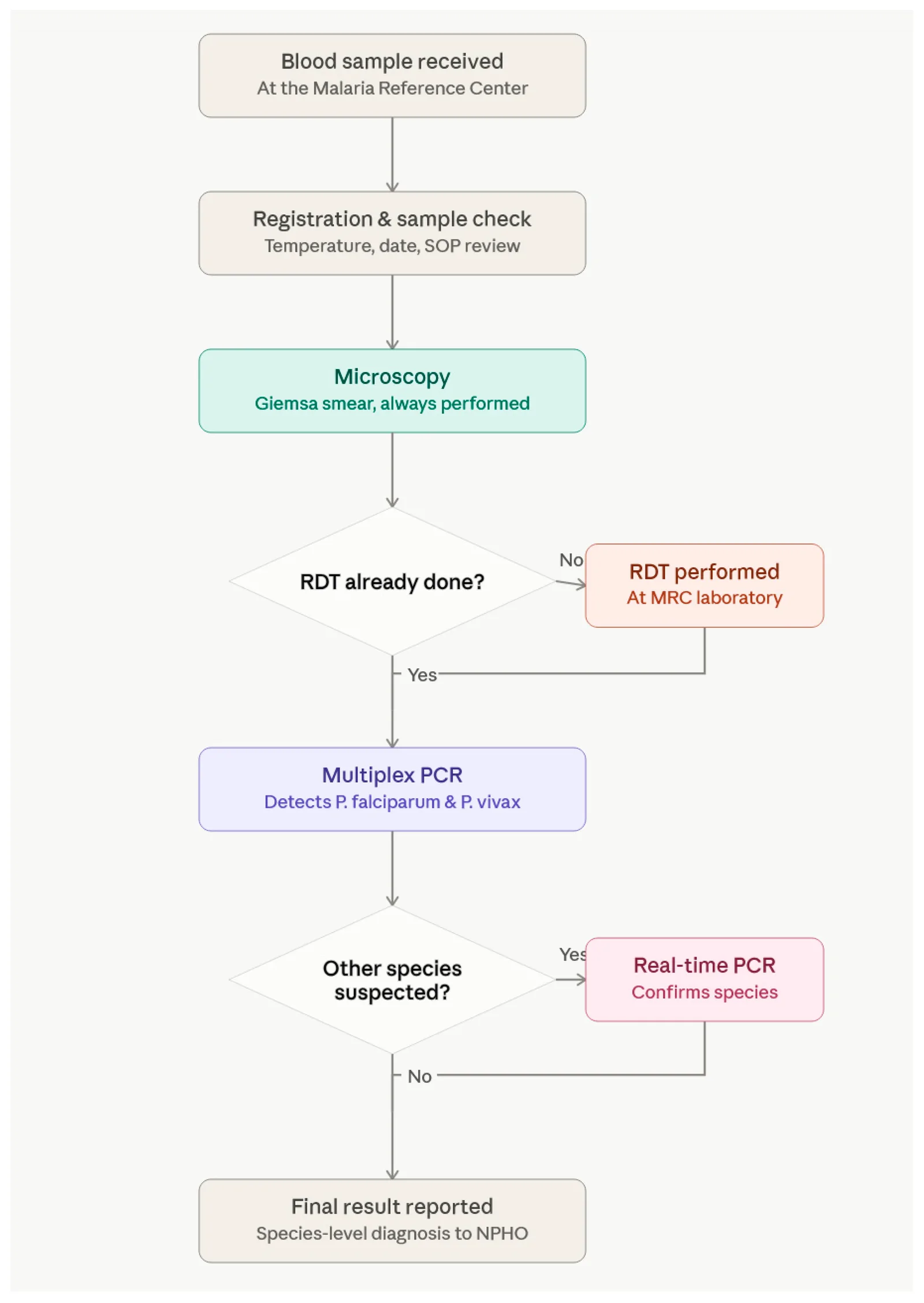
Recommended diagnostic workflow followed by MRC for suspected malaria cases. Abbreviations: SOP—standard operating procedure; RDT—rapid diagnostic test; PCR—polymerase chain reaction; MRC—Malaria Reference Center; NPHO—National Public Health Organization.

The Malaria Reference Center (MRC) in Greece has been accredited since 2015 (Hellenic Accreditation System: 1136), enabling malaria typing and *Plasmodium* sp. identification in suspected malaria cases. In this context, three different laboratory protocols have been accredited, namely microscopy, RDT, and PCR. The suggested diagnostic workflow is presented in Figure 1 and includes the initial implementation of microscopy, along with two Giemsa-stained thin films, examined independently by two different microscopists at approximately 200 fields before a slide is declared negative. At the same time, RDT examination is implemented at the MRC, provided that the referral hospital has not already performed the same test. In the latter case, the result of the RDT examination of the hospital is taken into consideration, since the RDTs provided by NPHO to the Greek hospitals have a similar performance for *P. falciparum* and *P. vivax* to the RDTs used by the MRC. The last step includes implementation of an in-house multiplex PCR protocol for detection in the same reaction of *P. falciparum* and *P. vivax* (taking into consideration that the majority of malaria cases in Greece fall within these two specific *Plasmodium* species).

Suspected malaria cases declared positive by microscopy as *P. ovale*, *P. malariae*, and *P. knowlesi* are verified using targeted species-level real-time PCR protocols, as described in a previous publication [16].

## 3. Results

### 3.1. Descriptive Analysis

A total of 780 clinical samples were checked for inclusion in the current retrospective analysis, with the majority of specimens obtained from whole blood. From those, a subset of 532 samples fulfilled the eligibility criteria (i.e., non-*P.ovale*, non-*P.malariae*, non-co-infection), with a valid result from all three different methods (PCR, microscopy, RDT). Multiplex polymerase chain reaction (PCR), which served as the reference standard methodology, identified 306 negative (57.5%) and 226 positive (42.5%) samples. Among positive PCR results, *P. falciparum* was the most common species detected (131 samples, 58%), followed by *P. vivax* (95 samples, 42%). Microscopy identified 326 negative (61.3%) and 206 positive (38.7%) samples. Among positive microscopy results, *P. falciparum* (111 samples, 54.0%) and *P. vivax* (95 samples, 46.0%) predominated. Furthermore, a total of 12 samples were positive for *P. ovale* and six samples were positive for *P. malariae*, and, in a single sample, a dual infection with *P. falciparum* and *P. vivax* was recorded. Since the abovementioned 19 samples could not be tested with all three implemented methodologies, they were excluded from further statistical analysis. For the *P. ovale*- and *P. malariae*-positive samples, verification at the species level was confirmed by the implementation of targeted species-level real-time PCR protocols.

The RDT identified 300 negative (56.4%) and 232 positive (43.6%) samples. Among positive RDT results, 135 samples (58.2%) were identified as *P. falciparum* via the HRP2 antigen line, while 97 samples (41.8%) tested positive for the pan-malarial pLDH antigen (“Pan”), consistent with non-*falciparum* infection. The distribution of detailed test results across the three different diagnostic methods (PCR, microscopy and RDT) is presented in Table 1. As mentioned in the Materials and Methods Section, the Pan results from RDT were mapped as positive findings for *P. vivax* in order to be able to perform a species-level agreement analysis between PCR, microscopy and RDT.

### 3.2. Binary Agreement with Reference Method

**Figure 2:**
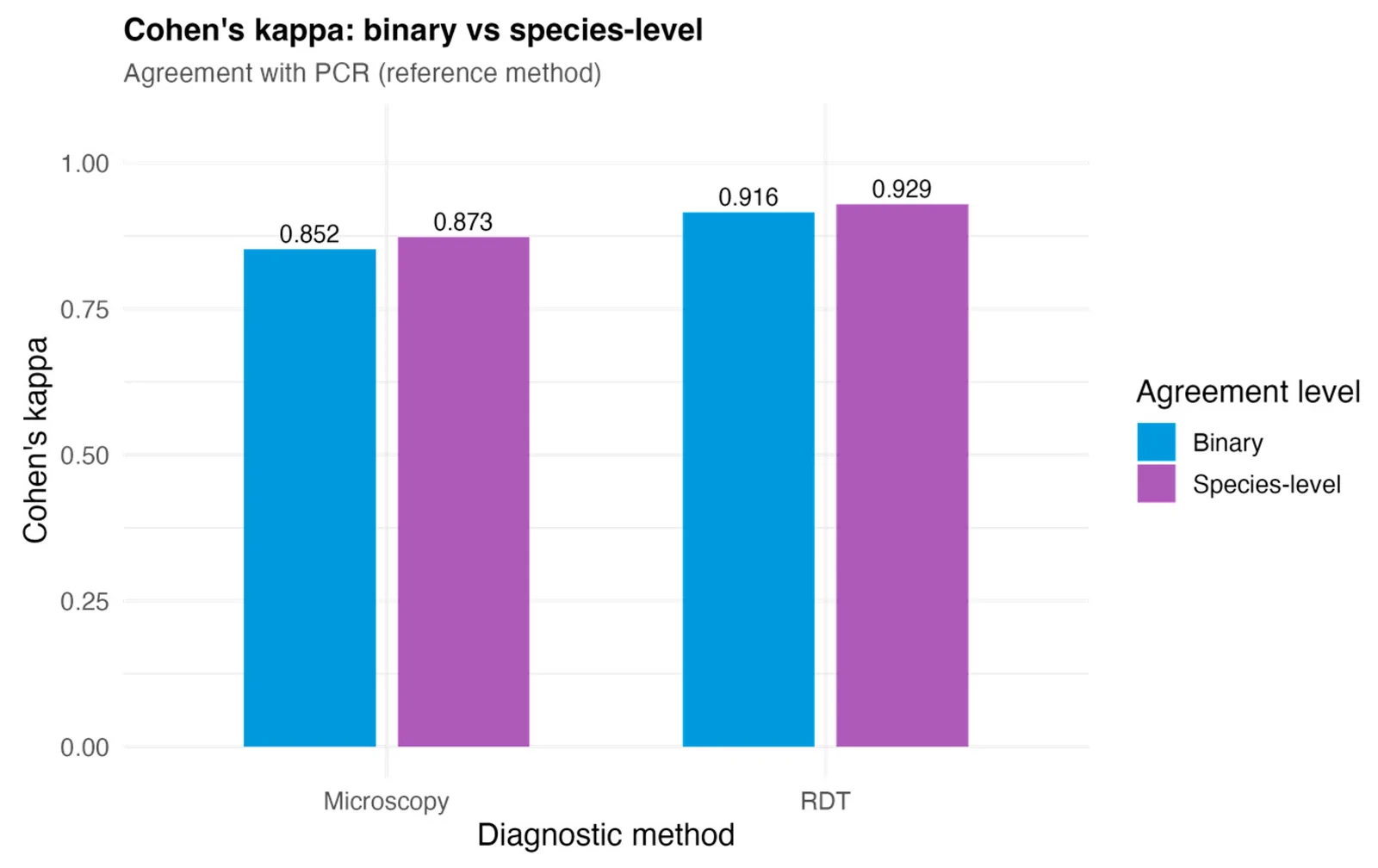
Cohen’s κ values for RDT and microscopy, as opposed to PCR, under two classification schemes: binary (positive vs. negative) and multicategory (species-level). Each bar shows the κ statistic (with 1.00 being perfect agreement) for one method. Blue bars correspond to binary diagnostic agreement, and purple bars correspond to species-level agreement. Abbreviations: RDT—rapid diagnostic test; PCR—polymerase chain reaction.

When evaluated against PCR as the reference method, microscopy achieved a sensitivity of 87.2% (95% CI: 82.2–90.9%) and specificity of 97.1% (95% CI: 94.5–98.4%), with a PPV of 95.6% (95% CI: 91.9–97.7%) and NPV of 91.1% (95% CI: 87.5–93.7%), based on 532 samples with valid results for both methods. The corresponding Cohen’s κ was 0.852 (95% CI: 80.6–89.5%), indicating strong agreement with PCR. The RDT, evaluated across 532 samples, achieved a sensitivity of 96.5% (95% CI: 93.2–98.2%) and specificity of 95.4% (95% CI: 92.5–97.3%), with a PPV of 94.0% (95% CI: 90.1–96.4%) and an NPV of 97.3% (95% CI: 94.8–98.6%). The Cohen’s κ for RDT was 0.916 (95% CI: 87.9–94.7%), reflecting an almost perfect agreement with PCR. These binary performance metrics are summarized in Table 2, and the κ values for both binary and species-level agreement are illustrated in Figure 2.

### 3.3. Species-Level Agreement and Misclassification Patterns

**Figure 3:**
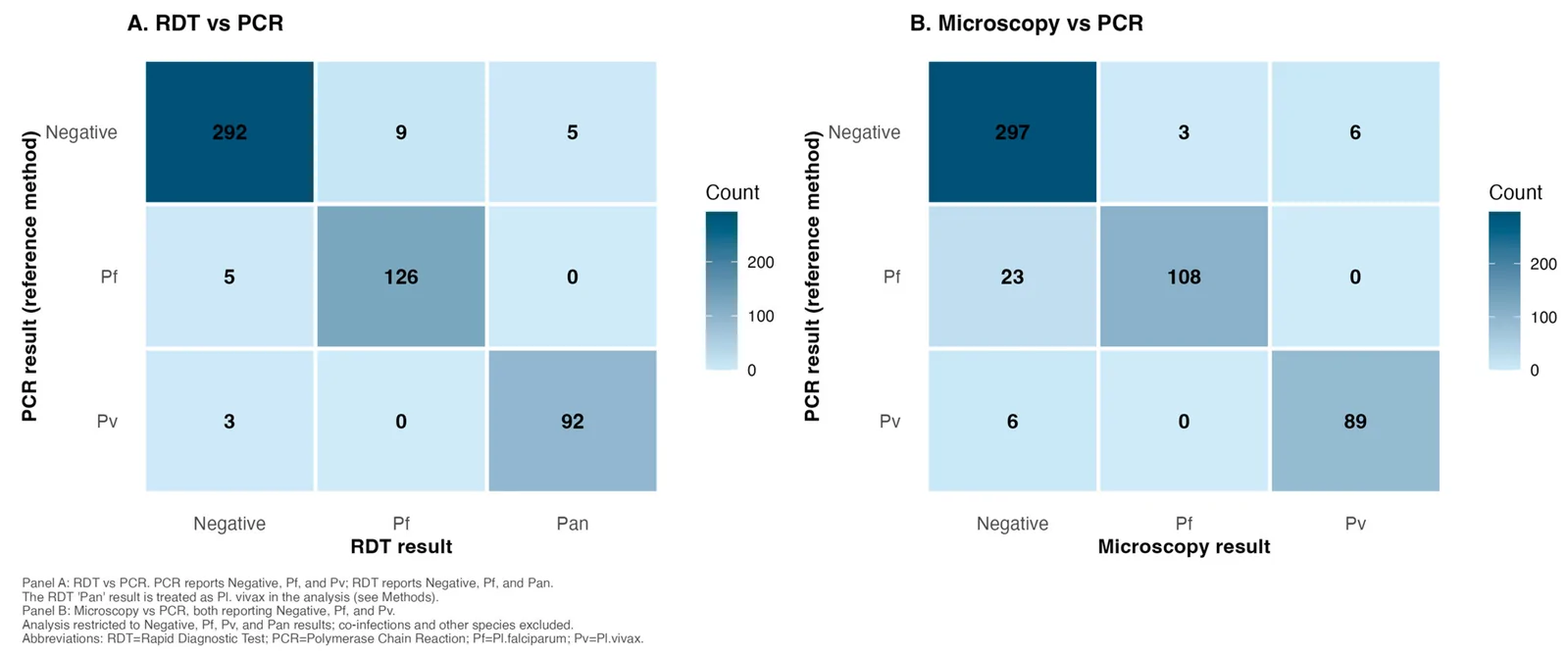
Confusion matrices of microscopy and RDT vs. PCR (adjusted for species grouping). (**A**) shows RDT vs. PCR (with RDT results grouped as negative, Pan, and Pf and PCR results as negative, Pf, and Pv). (**B**) shows microscopy vs. PCR (with microscopy species grouped as negative, Pf, and Pv and PCR results as negative, Pf, and Pv). Abbreviations: RDT—rapid diagnostic test; PCR—polymerase chain reaction; Pf—*P. falciparum*; Pv—*P. vivax*.

Species-level agreement was assessed for each method (i.e., microscopy and RDT) relative to PCR. With the RDT pan-antigen (“Pan”) result treated as *P. vivax*, all three methods shared a common three-category classification (negative, *P. falciparum*, *P. vivax*), and confusion matrices were constructed accordingly and presented in Figure 3.

Microscopy achieved a species-level Cohen’s κ of 0.873 (95% CI: 0.832–0.910), reflecting strong agreement with PCR (Figure 2). The confusion matrix confirmed a clear diagonal pattern, with 108 *P. falciparum* and 89 *P. vivax* samples correctly identified by both methods, alongside 297 concordant negatives. The principal source of discordance was a subset of PCR-positive *P. falciparum* samples that returned negative on microscopy (23 samples), consistent with submicroscopic parasitemia or post-treatment sampling; a further six PCR-positive *P. vivax* samples were likewise missed by microscopy. Off-diagonal entries in the opposite direction were rare, comprising only nine PCR-negative samples in which microscopy reported a species (three *P. falciparum* and six *P. vivax* samples). Notably, no *P. falciparum* sample was misidentified as *P. vivax* by microscopy or vice versa.

For the RDT, species-level agreement with PCR yielded a κ of 0.929 (95% CI: 0.896–0.958), indicating an almost perfect agreement. This value is closely comparable to the RDT’s binary κ (0.916), reflecting the fact that, once *P. ovale* and *P. malariae* are excluded, the pan-pLDH line reliably captures *P. vivax* infections. The confusion matrix illustrates this: of 95 PCR-confirmed *P. vivax* samples, 92 triggered the pan-pLDH line and were correctly reported as Pan-positive, while three were missed entirely. Not a single *P. vivax* sample was incorrectly identified as “Pf” by the RDT. Among PCR-confirmed *P. falciparum* samples, 126 were correctly identified via the HRP2 line, with five false-negative results. The off-diagonal entries among PCR-negative samples comprised nine Pf-positive and five Pan-positive RDT results, yielding a total of 14 false positives. This pattern is typically attributable to antigen persistence following treatment, a recognized feature of both HRP2-based and pLDH-based assays, whereby circulating antigen remains detectable after parasite clearance.

### 3.4. Species-Specific Diagnostic Performance

**Figure 4:**
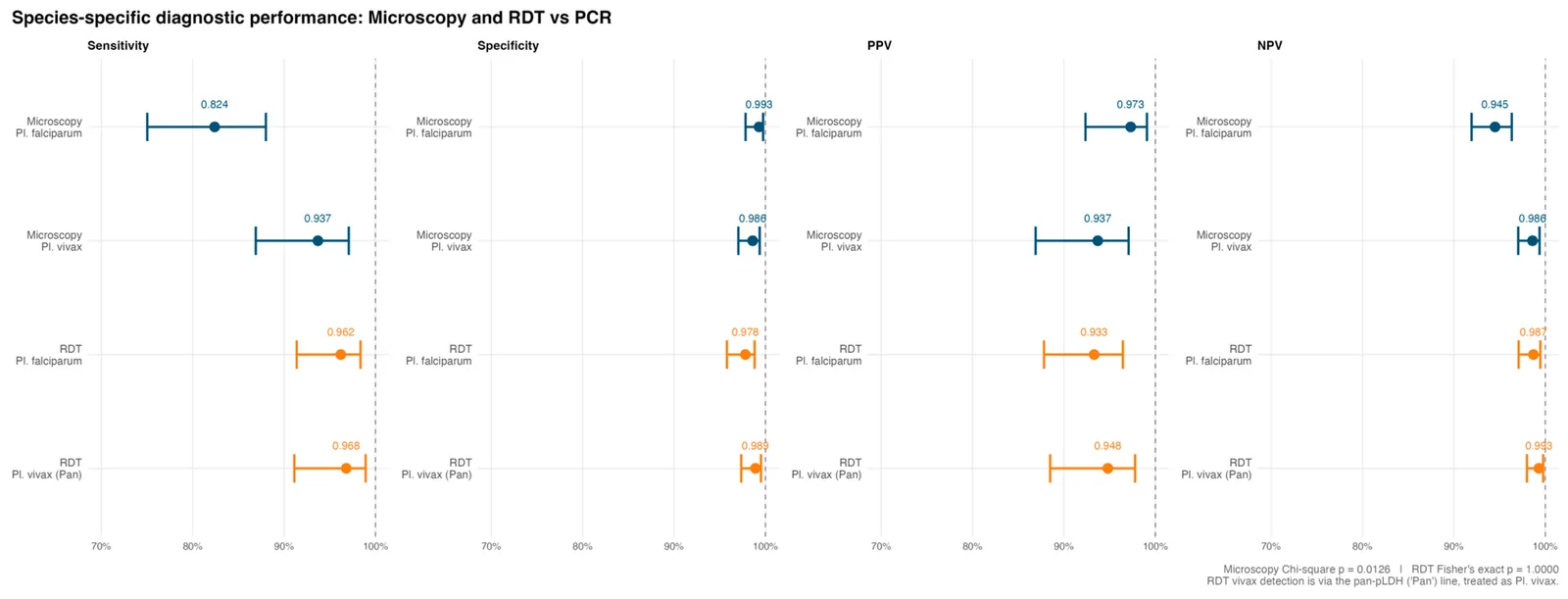
Diagnostic accuracy of microscopy and RDT for *Plasmodium* species using PCR as reference standard (point estimates with 95% Wilson confidence intervals; microscopy: Pf and Pv analyzed using one-versus-rest approach; RDT: Pf detected via HRP2 antigen line; non-Pf detected via pan-pLDH antigen line). Abbreviations: PCR—polymerase chain reaction; RDT—rapid diagnostic test; PPV—positive predictive value; NPV—negative predictive value.

Species-specific diagnostic performance was evaluated using a one-versus-rest approach, with results summarized in Figure 4.

Regarding the microscopic approach, sensitivity differed markedly between the two main *Plasmodium* species. The detection of *P. vivax* was highly reliable, with a sensitivity of 93.7% (95% CI: 86.9–97.1%) and specificity of 98.6% (95% CI: 97.0–99.4%), yielding a PPV and NPV of 93.7% (86.9–97.1%) and 98.6% (97.0–99.4%), respectively, a symmetric result reflecting that *P. vivax* misclassification was equally rare in both directions. The detection of *P. falciparum* by microscopy was notably lower, with a sensitivity of 82.4% (95% CI: 75.0–88.0%), while specificity remained very high at 99.3% (97.8–99.7%). The corresponding PPV was 97.3% (92.4–99.1%) and NPV was 94.5% (91.9–96.3%), indicating that, when microscopy reported *P. falciparum*, it was almost invariably correct, but a limited proportion of PCR-confirmed *P. falciparum* cases were not detected. This difference in sensitivity between *P. falciparum* and *P. vivax* was statistically significant (χ^2^ = 6.22, *p* = 0.013).

The RDT approach demonstrated consistently high sensitivity for both analytic targets. For *P. falciparum* detection via the HRP2 line, sensitivity was 96.2% (95% CI: 91.4–98.4%) and specificity was 97.8% (95.8–98.8%), with a PPV of 93.3% (87.8–96.5%) and NPV of 98.7% (97.1–99.5%). For non-Pf detection via the pan-pLDH line, sensitivity was 96.8% (91.1–98.9%) and specificity was 98.9% (97.3–99.5%). The PPV for non-Pf detection was 94.8% (88.5–97.8%), while the five false-positive Pan results observed in PCR-negative samples were consistent with antigen persistence (as discussed in Section 3.3). The NPV for non-Pf detection was 99.3% (98.0–99.8%). No statistically significant difference in sensitivity was found between the RDT’s Pf and non-Pf detection (Fisher’s exact test, *p* > 0.05).

## 4. Discussion

Malaria continues to be considered a major and persistent public health issue, threatening endemic but also non-endemic vulnerable and receptive countries around the world. In malaria-free countries, the majority of recorded cases are epidemiologically classified as imported, whereas transmission events are rarely recorded [17]. An updated travel history note is imperative for febrile travelers returning from malaria-endemic countries, constituting the prime target group for diagnosis of the disease [18]. Clinical presentation may lack specific clinical signs or symptoms, further necessitating the implementation of prompt and reliable diagnostic tools to ensure early and accurate diagnosis. Furthermore, a vast range of different diagnostic strategies is recorded between medical laboratories, especially in non-endemic countries, further challenging the diagnostic approach [19].

Accredited clinical laboratories implement targeted methodologies in order to ensure the validity of diagnostic results. Towards that direction, the MRC in Greece implements a triad of methodologies (including microscopy, RDT and PCR) for the typing and *Plasmodium* species identification of suspected malaria cases. The results of the current seven-year retrospective study verified the validity of microscopy as a gold standard laboratory approach in terms of proper detection and identification at the species level (i.e., *P. falciparum*, *P. vivax*, *P. ovale* and *P. malariae*). Furthermore, the validity of the implemented multiplex PCR protocol, an in-house protocol set by the personnel of the MRC, was also corroborated by the successful identification of *P. falciparum* and *P. vivax* cases, which comprised the majority of investigated clinical samples [13]. Hence, PCR can reliably assist microscopy in credible identification at the species level, especially in low parasitemia cases, due to its high diagnostic efficiency as a laboratory protocol. The validity of the RDT approach was also highlighted in the current study, resulting in the successful identification of *P. falciparum* cases, with the limitations of the method for the identification of non-falciparum species being well described in the literature [20,21]. For a number of different clinical samples, the MRC did not perform an RDT due to the fact that the majority of hospitals referring patient cases to MRC had already performed that method in the clinical sample that was subsequently sent for final verification of malaria typing and *Plasmodium* species identification. Since NPHO provides the same RDT for referring hospitals and MRC (Abbott, Bioline), the RDT of the referring hospital is recorded, and the other two remaining methods (i.e., PCR and microscopy) are implemented by MRC.

The experience of the MRC in malaria typing and *Plasmodium* species identification, accredited since 2015, has shown that the vast majority of malaria clinical cases diagnosed in Greece fall within two *Plasmodium* species, namely *P. falciparum* and *P. vivax*. This fact justifies the choice of the implementation of the multiplex PCR protocol targeting these two specific *Plasmodium* species. In terms of the *P. falciparum*-positive samples, PCR succeeded in identifying cases that were referred for diagnostic evaluation after the initiation of malaria treatment. In these samples, no *Plasmodium* parasites were identified by microscopy, since antimalarial regimens eradicate parasites; however, PCR protocols are characterized by increased sensitivity levels, enabling the detection of even residual copies of *P. falciparum* parasites. Towards that direction, PCR provides a necessary tool for ensuring successful *Plasmodium* species identification for malaria cases sampled after receiving treatment, along with cases of submicroscopic parasitemia [22,23]. Microscopy and PCR exhibited an almost complete agreement in terms of successfully identifying *P. vivax*-positive cases, thereby confirming the validity of the implemented molecular protocol for the second most commonly diagnosed *Plasmodium* species in Greece [13].

Amongst the total number of the clinical samples tested in the MRC with microscopy and PCR, a single case of dual infection with *P. falciparum* and *P. vivax* was recorded. This sample was successfully identified by PCR, giving the expected PCR products in electrophoresis (namely 346 bp for *P. falciparum* and 266 bp for *P. vivax*). Microscopy correctly identified that same sample due to the disproportionately increased parasitemia rate of *P. falciparum* hindering the presence of *P. vivax*. It has to be mentioned that identification at the species level was achieved due to the expertise and skills of the MRC personnel [24].

A small number of the tested clinical samples of the study were found positive for *P. ovale* and *P. malariae* through microscopy. According to the diagnostic workflow suggested by MRC, these species were verified by implementation of targeted to the species-level real-time PCR protocols. This approach has been successfully tested by the MRC, as described in a previous publication [16].

A series of limitations were recorded in different clinical samples during the experimental phase of the current study. More specifically, doubtful and unsuitable samples were excluded from the implemented statistical analysis. As far as the term doubtful sample is concerned, microscopy samples were selected that included destroyed morphological stages of *Plasmodium*, thereby not enabling identification at the species level. As far as the term unsuitable sample is concerned, samples were selected that did not fulfill the SOPs set by MRC (i.e., insufficient sample volume, non-febrile blood sample, clotted blood sample), and these samples were therefore excluded from the current study.

The implementation of binary agreement analysis resulted in an almost perfect agreement κ value for RDT and a strong agreement κ value for microscopy, as opposed to PCR being the reference standard methodology. Furthermore, the implementation of species-level agreement analysis (multicategory outcome) resulted in an almost perfect agreement κ value for RDT and a strong agreement κ value for microscopy for *P. falciparum* and *P. vivax* samples. This type of statistical analysis revealed certain expected limitations relating to the nature of microscopy and RDT, which have been verified by other studies as well [10,25,26]. Namely, samples positive for *P. falciparum* on PCR could not be detected by microscopy, including either submicroscopic or post-treatment clinical cases.

Greece, a non-endemic malaria country, faces diagnostic challenges regarding the surveillance of malaria. Unlike endemic settings, where the majority of cases can be attributed to only two species, either *P. falciparum* or *P. vivax*, laboratories in non-endemic countries must remain prepared to promptly and accurately identify all five *Plasmodium* species infecting humans, namely *P. falciparum*, *P. vivax*, *P. malariae*, *P. ovale*, and *P. knowlesi*, along with potential cases of dual infection. This situation is further complicated by the increasing number of imported cases from travelers and migrants arriving from diverse endemic regions, as well as by sporadic locally acquired cases, introducing a wider and less predictable range of malaria cases that would typically be encountered in an endemic setting. Consequently, non-endemic reference centers, such as the Greek Malaria Reference Center, cannot rely on a narrow, high-probability diagnostic algorithm targeted at one or two dominant species, but must instead maintain a comprehensive diagnostic capacity, combining microscopy, rapid diagnostic testing, and sensitive and specific molecular methods, to ensure that rare or morphologically ambiguous species are not overlooked or missed.

In summary, the results of this study are in accordance with other studies highlighting the essence of microscopic examination in malaria diagnostic procedures aimed at typing and *Plasmodium* species identification. Submicroscopic cases of clinical samples, along with clinical samples having received an antimalarial therapeutic scheme, can be handled through use of a targeted molecular diagnostic tool, as in the multiplex PCR approach suggested by the Greek MRC. The combination of microscopy, PCR, and the contribution of RDT is a credible three-way approach, ensuring the validity and reproducibility of extracted laboratory data in a certified laboratory diagnosing clinical samples suspected for malaria. Furthermore, clinical samples found to be positive for *P. ovale*, *P. malariae*, or *P. knowlesi* during microscopy can be verified through a combination of microscopy and real-time PCR, as suggested by the recommended diagnostic workflow.

## Figures and Tables

**Table 1:** Distribution of test results across implemented diagnostic methods. Abbreviations: RDT—rapid diagnostic test; PCR—polymerase chain reaction; * Pan result from RDT will be considered as positive finding for *P. vivax*.

Category	PCR	Microscopy	RDT
Negative	306 (57.5%)	326 (61.3%)	300 (56.4%)
*P. falciparum*	131 (58.0%)	111 (54.0%)	135 (58.2%)
*P. vivax*	95 (42.0%)	95 (46.0%)	97 (41.8%) *
TOTAL	532 (100%)	532 (100%)	532 (100%)

**Table 2:** Binary diagnostic performance of microscopy and RDT against PCR as reference method. Sensitivity, specificity, PPV, and NPV presented with 95% Wilson confidence intervals. Kappa with 95% bootstrap confidence interval (B = 2000 resamples). N reflects pairwise valid results for each method vs. PCR. Abbreviations: TP—true positive; TN—true negative; FP—false positive; FN—false negative; PPV—positive predictive value; NPV—negative predictive value; RDT—rapid diagnostic test; Sen—sensitivity; Spe—specificity.

Method	N	TP	TN	FP	FN	Sen (95% CI)	Spe (95% CI)	PPV (95% CI)	NPV (95% CI)	Kappa (95% CI)
Microscopy	532	197	297	9	29	0.872 (0.822–0.909)	0.971 (0.945–0.984)	0.956 (0.919–0.977)	0.911 (0.875–0.937)	0.852 (0.806–0.895)
RDT	532	218	292	14	8	0.965 (0.932–0.982)	0.954 (0.0.925–0.973)	0.940 (0.901–0.964)	0.973 (0.948–0.986)	0.916 (0.879–0.947)

## Data Availability

The original contributions presented in the study are included in the article; further inquiries can be directed to the corresponding author.

## References

[1] Anstey N.M., Grigg M.J. (2019). Zoonotic Malaria: The Better You Look, the More You Find. J. Infect. Dis..

[2] WHO (2025). World Malaria Report 2025. https://www.who.int/teams/global-malaria-programme/reports/world-malaria-report-2025.

[3] Crutcher J.M., Hoffman S.L. (1996). Chapter 83. Malaria. Medical Microbiology.

[4] Mbanefo A., Kumar N. (2020). Evaluation of Malaria Diagnostic Methods as a Key for Successful Control and Elimination Programs. Trop. Med. Infect. Dis..

[5] NPHO (2025). Ετήσια έκθεση επιδημιολογικής επιτήρησης ελονοσίας, Ελλάδα, έτος 2025 (15 Pages). https://www.eody.gov.gr/images/Έκθεση_επιδημιολογικής_επιτήρησης_ελονοσίας_στην_Ελλάδα_2025.pdf.

[6] Malaria Microscopy Quality Assurance Manual. Version 2. https://www.who.int/docs/default-source/documents/publications/gmp/malaria-microscopy-quality-assurance-manual.pdf.

[7] Malaria. CDC Yellow Book (23 April 2025). https://www.cdc.gov/yellow-book/hcp/travel-associated-infections-diseases/malaria.html.

[8] WHO Guidelines for Malaria (13 August 2025). https://www.severemalaria.org/sites/default/files/content/document/WHO%20malaria%20guidelines%20-%20August%202025.pdf.

[9] Global Malaria Programme. Rapid Diagnostic Tests. https://www.who.int/teams/global-malaria-programme/case-management/diagnosis/rapid-diagnostic-tests.

[10] Calderaro A., Montecchini S., Buttrini M., Piccolo G., Rossi S., Arcangeletti M.C., Farina B., De Conto F., Chezzi C. (2021). Malaria Diagnosis in Non-Endemic Settings: The European Experience in the Last 22 Years. Microorganisms.

[11] Chavalitshewinkoon-Petmitr P. (2010). Laboratory diagnosis of malaria. Siriraj. Med. J..

[12] WHO (Global Malaria Programme) Technical Information. https://www.who.int/teams/global-malaria-programme.

[13] Patsoula E., Spanakos G., Sofianatou D., Parara M., Vakalis N.C. (2003). A single-step, PCR-based method for the detection and identification of *Plasmodium vivax* and *P. falciparum*. Ann. Trop. Med. Parasitol..

[14] McHugh M.L. (2012). Interrater reliability: The kappa statistic. Biochem. Med..

[15] Landis J.R., Koch G.G. (1977). The measurement of observer agreement for categorical data. Biometrics.

[16] Tegos N., Goumenopoulos C., Mpimpa A., Papavasilopoulos V., Beleri S., Patsoula E. (2024). Sensitivity Assessment of a Multiplex and Real-Time PCR Protocols for the Detection of malaria in External Quality Control Samples in the Malaria Reference Center in Greece. Parasitologia.

[17] Askling H.H., Bruneel F., Burchard G., Castelli F., Chiodini P.L., Grobusch M.P., Lopez-Vélez R., Paul M., Petersen E., Popescu C. (2012). European Society for Clinical Microbiology and Infectious Diseases Study Group on Clinical Parasitology. Management of imported malaria in Europe. Malar. J..

[18] The ‘World Malaria Report 2019′ at a Glance (4 December 2019). https://www.who.int/news-room/feature-stories/detail/world-malaria-report-2019.

[19] Agence Nationale de Sécurité du Médicament et des Produits de Santé (ANSM) Contrôle National de Qualité des Analyses de Biologie Médicale (30 November 2020–25 June 2021). https://ansm.sante.fr/documents/reference/controle-national-de-qualite-des-analyses-de-biologie-medicale.

[20] Maltha J., Gillet P., Jacobs J. (2013). Malaria rapid diagnostic tests in travel medicine. Clin. Microbiol. Infect..

[21] Moody A. (2002). Rapid diagnostic tests for malaria parasites. Clin. Microbiol. Rev..

[22] Ponce C., Kaczorowski F., Perpoint T., Miailhes P., Sigal A., Javouhey E., Gillet Y., Jacquin L., Douplat M., Tazarourte K. (2017). Diagnostic accuracy of loop-mediated isothermal amplification (LAMP) for screening patients with imported malaria in a non-endemic setting. Parasite.

[23] Bousema T., Okell L., Felger I., Drakeley C. (2014). Asymptomatic malaria infections: Detectability, transmissibility and public health relevance. Nat. Rev. Microbiol..

[24] Fitri L.E., Widaningrum T., Endharti A.T., Prabowo M.H., Winaris N., Nugraha R.Y.B. (2022). Malaria diagnostic update: From conventional to advanced method. J. Clin. Lab. Anal..

[25] Kavanaugh M.J., Azzam S.E., Rockabrand D.M. (2021). Malaria Rapid Diagnostic Tests: Literary Review and Recommendation for a Quality Assurance, Quality Control Algorithm. Diagnostics.

[26] Tseroni M., Pervanidou D., Tserkezou P., Rachiotis G., Pinaka O., Baka A., Georgakopoulou T., Vakali A., Dionysopoulou M., Terzaki I. (2015). Field application of SD Bioline Malaria Ag Pf/Pan rapid diagnostic test for Malaria in Greece. PLoS ONE.

